# Chromosomes of *Lepidochitona caprearum* (Scacchi, 1836) (Polyplacophora, Acanthochitonina, Tonicellidae) provide insights into Acanthochitonina karyological evolution

**DOI:** 10.3897/CompCytogen.v6i4.3722

**Published:** 2012-12-12

**Authors:** Agnese Petraccioli, Nicola Maio, Gaetano Odierna

**Affiliations:** 1 Dipartimento di Biologia Strutturale e Funzionale, Università di Napoli Federico II, Via Cinthia, 80126 Napoli, Italia; 2 Museo Zoologico, Centro Museale,Università di Napoli Federico II, via Mezzocannone 8, 80134 Napoli, Italia

**Keywords:** Chromosome evolution, chromosome banding, heterochromatin, chitons, *Lepidochitona caprearum*, Acanthochitonina

## Abstract

We describe the karyotype, location of nucleolus-organizing regions (NORs) and heterochromatin composition and distribution in *Lepidochitona caprearum* (Scacchi, 1836). The examined specimens had 2n=24 chromosomes; the elements of pairs 1–4 were metacentric, subtelocentric those of the fifth pair, telocentric the elements of other pairs. NOR-FISH, Ag-NOR- and CMA_3_ banding showed NORs localized on pericentromeric regions of a medium small sized, telocentric chromosome pair. After C-banding or digestions with restriction enzyme NOR associate heterochromatin only was cytologically evident, resulting CMA_3_ positive. The comparison with chromosome data of other chitons, other than to evidence a karyotypic similarity of *Lepidochitona caprearum* to species of suborder Acanthochitonina, allows us to infer that chromosome evolution in the suborder mainly occurred via reduction of the number of the chromosomes by centric fusions, which took place repeatedly and independently in the different lineages of Acanthochitonina.

## Introduction

Polyplacophora, known also as chitons, includes about 900 living species, exclusively marine, distributed worldwide, mostly from the intertidal to the sub-littoral zone (Slieker 2000). These mollusks are scarcely investigated from a karyological point of view: data are available for only 21 species, all of the order Chitonida (*sensu* Sirenko 2006), namely ten of the suborder Chitonina (six species of the family Chitonidae and four of Ischnochitonidae) and eleven of the suborder Acanthochitonina (seven species of Acanthochitonidae, three of Mopaliidae and one of Tonicellidae) (Table 1). Though few, the karyological data have provided valuable information for systematics and phylogeny of chitons (Odierna et al. 2008). In order to increase karyological data on this class of mollusks we performed a chromosomal analysis using both conventional and banding staining methods and in situ hybridization (NOR-FISH) on *Lepidochitona caprearum* (Scacchi, 1836). For this chiton karyological data concern the chromosome number of 2n=24 and some details on morphology of eight large elements (meta- or sub-metacentric) (Vitturi et al. 1982). Systematic and phylogenetic relationships of this species are debated. In addition, *Lepidochitona caprearum* has been the subject of several nomenclatural and taxonomic revisions. First Scacchi (1836) described this common Mediterranean chiton as *Chiton caprearum* Scacchi, 1836 (pag. 9); later, it was described by Reeve (1848) as *Chiton corrugatus* Reeve, 1848 (Plate 28, figure 185). Dall (1882) created the genus *Middendorffia* Dall, 1882 for it, and, successively, Kaas (1957) synonymised *Middendorffia caprearum* (Scacchi, 1836) with *Chiton corrugatus*. Successively, Kaas and van Belle (1981) carried out a systematic revision of perimediterranean and Atlantic species of the genus *Lepidochitona* Gray, 1821 and considered the taxon *Middendorffia* as synonym of the genus *Lepidochitona*. Finally, on the basis of the classification priority criterion, nomenclatural validity of the Scacchian taxon was demonstrated by Piani (1983) and a few years later by Gaglini (1985).

**Table 1.** Chomosome data of the chitons studied to date, classified according to Sirenko (2006). n= haploid number; FN = Fundamental number (arm number), M= metacentric, SM= Submetacentric, ST=subtelocentric; T=telocentric.

**Order**	**Suborder**	**Family**	**Species**	**n**	**Haploid chr. for.**	**FN**	Chitonida
Chitonida	Chitonina	Chitonidae	*Acanthopleura gemmata* (Blainville, 1825)	13	10 M, 3 SM	26	Yassen et al. (1995)
			*Chiton granosus* Frembly, 1827	12	6 M, 6 SM	24	Northland-Leppe et al. (2010)
			*Chiton kurodai* Is. & Iw. Taki, 1929	12	7 M, 4 SM, 1 ST	24	Yum and Choe (1996)
			*Chiton olivaceus* Spengler, 1797	13	12M, 1 SM	26	Vitturi et al. (1982)
			*Liolophura japonica* (Lischke, 1873)	12	12 M/SM	24	Nishikawa and Ishida (1969), Kawai (1976)
			*Onithochiton hirasei* Pilsbry, 1901	12			Nishikawa and Ishida (1969)
		Ischnochitonidae	*Ischnochiton boninensis* Bergenhayn, 1933	12			Nishikawa and Ishida (1969)
			*Ischnochiton comptus* (Gould, 1859)	12			Nishikawa and Ishida (1969)
			*Lepidozona albrechtii* (von Schrenck, 1862) [= *Tripoplax albrechtii* (von Schrenck, 1862) ]	12	10 M, 1M/SM, 1 SM	24	Choe et al. (1995), Yum and Choe (1996)
			*Lepidozona coreanica* (Reeve, 1847)	12	8 M, 1 M/SM, 3 SM	24	Nishikawa and Ishida (1969), Yum and Choe (1996)
	Acanthochitonina	Acanthochitonidae	*Acanthochitona achate*s (Gould, 1859)	8	5 M, 1 SM, 2 ST	16	Rho et al. (1998)
			*Acanthochitoa circellata* (A. Adams & Reeve MS, Reeve, 1847)	8	1 M, 4 SM, 2 ST, 1 T	15	Rho et al. (1998)
			*Acanthochitona communis* (Risso, 1826) [= A. fascicularis (Linnaeus, 1767)]	12	2M, 5T, ?	undefined	Vitturi et al. (1982)
			*Acanthochitona crinita* (Pennant, 1777)	9	5 M, 2 SM, 2 ST	18	Colombera and Tagliaferri (1983)
			*Acanthochitona defilippii* (Tapparone Canefri, 1874)	8	3 M, 3 SM, 1 ST, 1 T	15	Nishikawa and Ishida (1969),Kawai (1976), Rho et al. (1998)
			*Acanthochitona discrepans* (Brown, 1827)	9	7 M, 1 St, 1 T	17	Certain (1951) in Nishikawa and Ishida (1969)
			*Acanthochitona rubrolineata* (Lischke, 1873)	8	5 M, 1 SM, 1 SM/ST, 1 ST	15	Nishikawa and Ishida (1969), Rho et al. (1998)
		Mopaliidae	*Katharina tunica* (Wood, 1815)	6	4 M, 2 T	10	Dolph and Humphrey (1970)
			*Nuttallochiton mirandus* (E. A. Smith MS, Thiele, 1906)	16	1M, 1SM, 14T	18	Odierna et al. (2008)
			*Placiphorella stimpsoni* (Gould, 1859)	12	6 M, 1 ST, 5 T	19	Nishikawa and Ishida (1969), Yum and Choe (1996)
		Tonicellidae	*Lepidochitona caprearum* (Scacchi, 1836)	12	4M/SM, ?	undefined	Vitturi et al. (1982)
				12	4 M, 1 ST, 7 T	17	present paper

## Material and methods

We studied 4 males and 3 females of *Lepidochitona caprearum* from Seiano (Naples, Italy) and 3 males and two females from Gaeta (Latina, Italy).

Gonads of each individual were excised and incubated for two hours in 1 ml of calf serum, previously heat inactivated at 56°C for 30 min, containing 50 ml of colcemid at 10 mg/ml. Then, the gonads were incubated for 30 min in hypotonic solution (KCl 0.075 M + sodium citrate 0.5%, 1:1) and fixed for 15 min in methanol + acetic acid, 3:1. After that, cell dissociations of gonads were made on a tea steel sieve and 20 μl of cell suspensions were dropped on clean slides (Petraccioli et al. 2010).

Standard chromosome staining was performed by using 5% Giemsa, pH 7.0. The following chromosome banding techniques also were used: Ag-NOR staining of Nucleolus Organizer Regions (Ag-NORs), chromomycin A_3_ (CMA_3_)/ methyl green staining, quinacrine (Q) banding, DA/DAPI, C-banding and sequential staining of C-banding+CMA_3_+DAPI (details in Odierna et al. 2008), conducting the incubation in Ba(OH)_2_ for 2 min and at room temperature. Karyotypes were constructed from seven Giemsa-stained mitotic metaphase plates and used to measure chromosome centromeric index (CI) and relative length (RL) according to the nomenclature by Levan et al. (1964).

NOR-FISH was performed as described by Petraccioli et al. (2010), with slight modifications, using as probe PCR amplified and biotinaled 18S rRNA gene sequence units of the pectenid *Adamussium colbecki* (Smith, 1902). Slides were aged for a week at room temperature and two hours a 60°C, and then incubated for 30 min in Rnase at 100 mg/ml in Tris-HCl pH 6.5. Slides were washed two min for each ethanol 50, 70, 90 and 100% and air dried. Chromosomes and probe were denatured at 72°C with the hybridization mixture (10 ng/ml biotinylated 16 dUTP probe + 0.1 mg/ml shared *Escherichia coli* DNA in 2xSSC with 50% formamide) for 2 min. The hybridizations were carried over-night at 40°C. After washing in 1xSSC at 72°C for 5 min and at RT for 2 min in blocking solution (dry milk 2% + 0,1% of Tween 20 in 4xSSC), cytochemical detection was performed by incubating slides for 1 h with monoclonal anti-biotin (Sigma cod. B7653) diluted 1:500 in PTB (1 ml PTB= 5 μl of Tween 20% + 0.01 g of Dry milk + in 1 ml of PBS 0,2 M), washing in 1xPBS and incubating for 30 min in anti-anti-biotin diluted 1:50 in PTB. After washing in PBS, slides were counterstained with 5 μg/ml propidium iodide (PI) in 1xPBS for 15 min at room temperature and, finally, mounted with antifade (DABCO, Sigma). The hybridization signals were detected and recorded under an epifluorescent microscope (Axioscope Zeiss) equipped with a digital camera.

## Results

**Figure 1.**
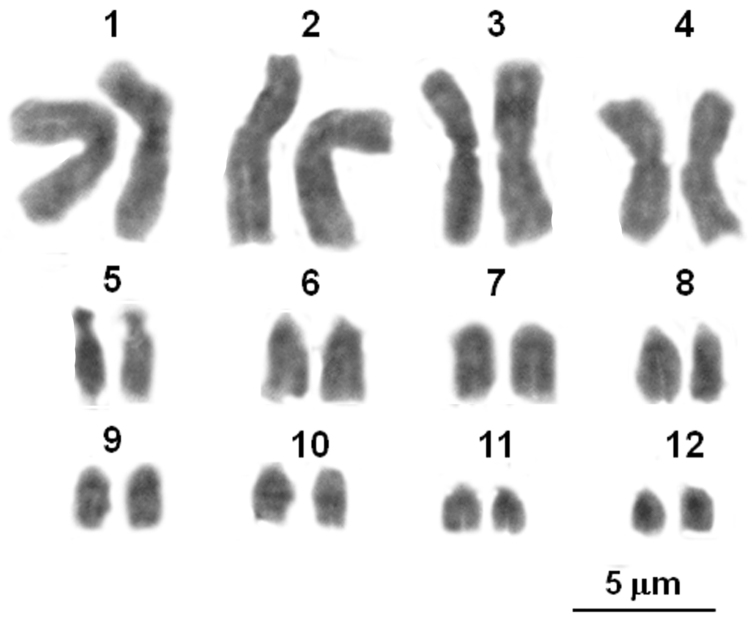
Giemsa stained karyotype of a male of *Lepidochitona caprearum* from Seiano (Naples, Italy).

**Figure 2.**
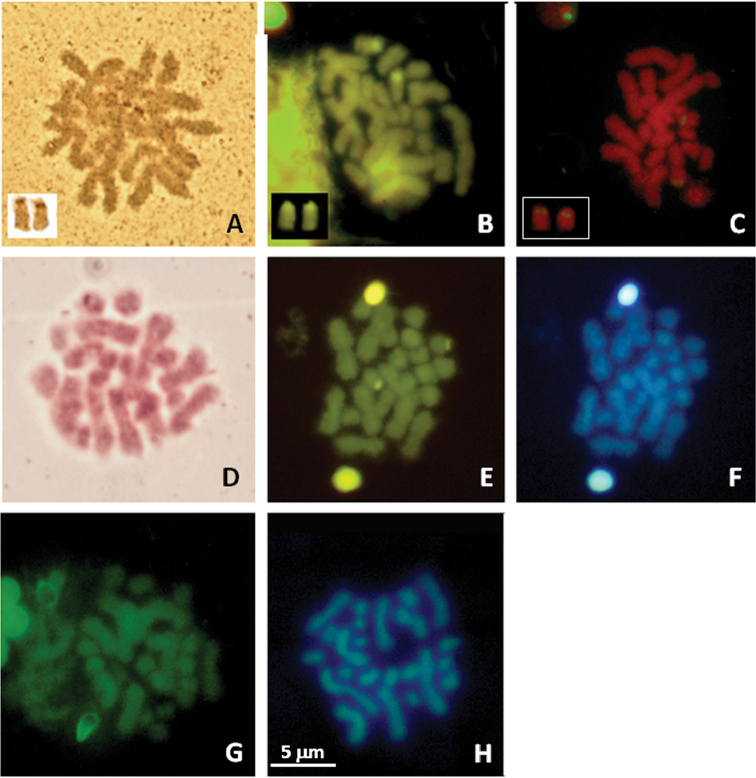
Male (**A**, **C**, **E**, **F** and **G**) from Seiano, Naples, Italy, and female, from Gaeta, Latina, Italy, (**B**, **D** and **H**) metaphase plates of *Lepidochitona caprearum*, stained with Ag-NOR banding (**A**),CMA_3_ banding (**B**), NOR-FISH (**C**), C-banding + Giemsa (**D**); C+banding + CMA_3_ (**E**)+DAPI (**F**), Quinacrine (**G**) and DA/DAPI (**H**). Panels in A, B and C include their relative NOR bearing chromosome pair. Scale bar in **H** refers all images.

Twelve bivalents, four larger than the other eight ones resulted present in 25 examined male, diakinetic, meiotic figures (Fig. 1). The diploid number of 2n=24 chromosomes was confirmed by the examination of 15 spermatogonial and ten oogonial metaphase plates. Independently of sex and provenance, karyotypes consisted of four pairs (1–4) with metacentric elements, a pair (the fifth) with subtelocentric chromosomes, the remaining pairs (6–12) included telocentric elements (haploid chromosome formula: 4M, 1ST, 7T; Arm number, FN=17 (Table 2; Fig. 1). One NOR bearing pair resulted evidenced after staining with Ag-NOR-, CMA_3_ banding and NOR-FISH; loci NORs were on pericentromeric regions of two medium sized telocentric chromosomes, tentatively the pair eight or nine (Fig. 2 A, B and C). After C-banding staining or digestions with Restriction enzyme *Alu*I, NOR associated heterochromatin only was well evident, resulting CMA_3_ positive and DAPI negative (Fig. 2 D, E and F). Quinacrine and DA/DAPI banding uniformly stained the chromosomes (Fig. 2 G and H).

**Table 2.** Chromosome morphometric parameters of *Lepidochitona caprearum*, according to Levan et al. (1964); M= metacentric, ST= subtelocentric, T= telocentric.

**Chromosome**	**Relative Length (RL) mean ± SD**	**Centromeric index (CI) mean ± SD**	**Chromosome type**
1	18.2 ± 0.5	48.3 ± 3.0	M
2	17.0 ± 0.7	39.9 ± 2.8	M
3	15.2 ± 0.4	49.0 ± 3.1	M
4	12.8 ± 0.6	39.1 ± 2.9	M
5	7.7 ± 0.5	18.2 ± 2.0	ST
6	6.2 ± 0.4	0	T
7	5.3 ± 0.3	0	T
8	4.0 ± 0.5	0	T
9	3.9 ± 0.6	0	T
10	3.8 ± 0.4	0	T
11	3.2 ± 0.5	0	T
12	2.7 ± 0.4	0	T

## Discussion

**Figure 3.**
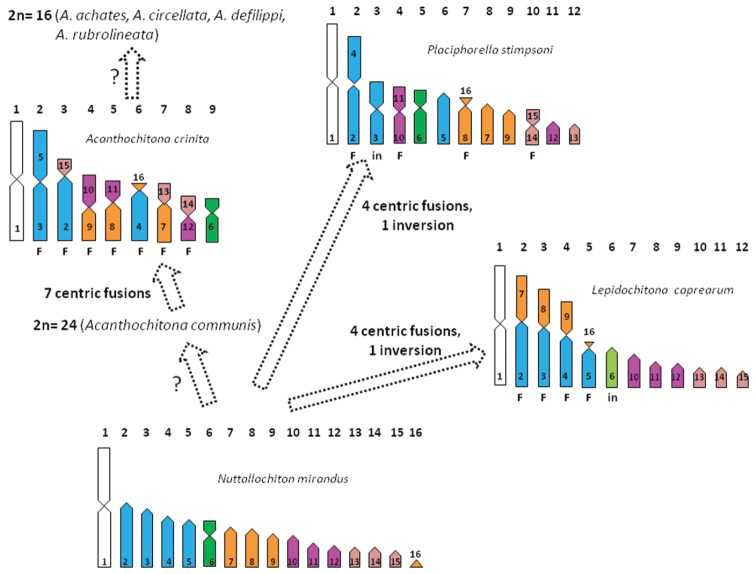
Hypothesis on the derivation of the karytotypes of *Lepidochitona caprearum*, *Placiphorella stimpsoni* and *Acanthochitona crinita* from that of *Nuttallochiton mirandus*. Haploid chromosome ideograms have been depicted according to the relative length and centromeric indexes given by Yum and Choe (1996) for *Placiphorella stimpsoni*, Colombera and Tagliaferri (1970) for *Acanthochitona crinita*, Odierna et al. (2008) for *Nuttallochiton mirandus* and the present paper for *Lepidochitona caprearum*. The numbers included in the chromosomes refer to those of *Nuttallochiton mirandus* supposed involved in the chromosome changes.

According to the classification by Kaas and Van Belle (1998) species of genus *Lepidochitona* belong to the family Ischnochitonidae Dall, 1989, suborder Chitonina. In contrast, Sirenko (2006), in his classification, included *Lepidochitona* in the family Tonicellidae Simroth, 1894, suborder Acanthochitonina. In agreement with Vitturi et al. (1982) we find that *Lepidochitona caprearum* possesses 2n=24 chromosomes. This chromosome number is also displayed by all the so far studied species of Ischnochitonidae, namely two species of *Ischnochiton* Gray, 1847 and two of *Lepidozona* Pilsbry,1892 (Nishikawa and Ishida 1969, Choe et al. 1995, Yum and Choe 1996). Only for the two *Lepidozona* species the chromosome morphology is given (Yum and Choe 1996), and in both cases the elements only are metacentric or submetacentric. This kind of chromosome sets can be ranked more or less symmetric (White 1978), that is karyotypes only including a series of elements gradually decreasing and with chromosome arms of almost equal length. Interestingly, the other so far investigated species of the suborder Chitonina possess karyotypes of 2n=24 or 26 elements metacentric or submetacentric, (see Table 1), excluding *Chiton kurodai* Is. & Iw. Taky, 1929, which has a karyotype with a pair of subtelocentric elements (Yum and Choe 1996). In contrast, even if possessing 2n=24 elements, the karyotype of *Lepidochitona caprearum* strongly deviates from those of Chitonina species. In fact, other than biarmed chromosomes, its karyotype includes also subtelocentric and telocentric elements. Interestingly, a similar karyotype is also displayed from all Acanthochitonina species (see Table 1), to which, then, *Lepidochitona caprearum* is karyologically related. Molecular phylogenetic study on chitons by Okusu et al. (2003) suggests a close relationship between *Lepidochitona* and the mopaliid species, *Katharina tunicata* (Wood, 1815), which, according to Dolph and Humphrey (1970), possesses 2n=12 chromosomes with a chromosome formula of 8M+4ST. However, both molecular relationship and chromosome record for *Katharina tunicata* have to be considered with caution. In fact, Mopaliidae in the molecular phylogeny by Okusu et al. (2003), appear polyphyletic, a state not considered in the systematic revision by Sirenko (2006), where Mopaliidae are monophyletic. Concerning chromosome data of *Katharina tunicata*, the record by Dolph and Humphrey (1970) needs confirmation, because from examination of the figure provided by the authors, all chromosome pairs are unpaired (each pair contains elements differing in length and/or shape). However, among Acanthochitonina a set with 2n=24 elements is shown by two species: one of the family of Acanthochitonidae, namely *Acanthochitona communis* (Risso, 1826) [= *Acanthochitona fascicularis* (Linnaeus, 1767)], but with the chromosome formula not completely resolved (Vitturi et al. 1982); the second species of the family of Mopaliidae, namely, *Placiphorella stimpson*i (Gould, 1859), which has a chromosome formula of 6M, 1ST, 5T (Yum and Choe 1996) (Table 1). However, the karyotypes of *Placiphorella stimpsoni* and *Lepidochitona caprearum* are strongly divergent (see Table 1). In fact their chromosome sets differ both in the number of metacentric and telocentric elements and because in the set of *Placiphorella stimpsoni* the first two pairs are markedly longer than the other pairs, while in *Lepidochitona caprearum* are four the pairs clearly longer than the other ones (see Fig. 3 for a comparison). So, multiple and complex chromosome rearrangements occur for the transition between karyotypes of *Lepidochitona caprearum* and *Placiphorella stimpsoni*. A possible, alternative scenario for the origin of their chromosome set is given in Fig. 3. The scenario is based on the hypothesis, that we advanced in our previous study (Odierna et al. 2008), according to which a karyotype like that of *Nuttallochiton mirandus* (E. A. Smith MS, Thiele, 1906), of 2n=32 elements with a chromosome formula of 1M, 1SM, 14T, is primitive and the karyotypes with lesser chromosome number derived from it, mainly by a series of Robertsonian fusions. Accordingly, the karyotype of *Lepidochitona caprearum* could have arisen from a *Nuttallochiton mirandus* like karyotype by four centric fusions plus one inversion (see Fig. 3). Similarly, one inversion and four centric fusions also could give rise to the karyotype of the *Placiphorella stimpsoni* from one *Nuttallochiton mirandus* like. In addition, a derivation from a karyotype *Nuttallochiton mirandus* like could also be supposed for that one of 2n=18 chromosomes of the Acanthochitonid species, *Acanthochitona crinita* (Pennant, 1777), (Colombera and Tagliaferri 1983): in fact, seven centric fusions occur for the transition from *Nuttallochiton mirandus* like karyotype to that of *Acanthochitona crinita* (see Fig. 3). Moreover, in this genus a further reduction to 2n=16 chromosomes also occurred; since this chromosome number is showed by *Acanthochitona achates* (Gould, 1859), *Acanthochitona circellata* (A. Adams & Reeve MS, Reeve, 1847) *Acanthochitona defilippi* (Tapparone Canefri, 1874), and *Acanthochitona rubrolineata* (Lischke, 1873) (see Table 1). Interestingly, in this genus the reduction of chromosomes number to 2n=18 or 16 an intermediate step of 2n=24 could not be ruled out, as suggested by the karyotype of *Acanthochitona communis*, which has 2n=24 elements (Vitturi et al. 1982). It should be noted that for the derivation of the chromosome set of *Lepidochitona caprearum*, *Placiphorella stimpsoni* and *Acanthochitona crinita*, different elements of the karyotype like that of *Nuttallochiton mirandu*s have supposedly been involved both in the centric fusions and inversions, meaning that these rearrangements have occurred repeatedly and independently in the diverse lineages of suborder Acanthochitonina. This hypothesis on the chromosome evolution in Acanthochitonina is also the most parsimonious and supports the inclusion of *Lepidochitona* in the suborder Acanthochitonina operated by Sirenko (2006) in his chiton systematic revision.

Studies on NOR localization and heterochromatin distribution and composition proved to be valuable in providing taxonomic, systematic and evolutionary information in several taxa, including bivalves (Thiriot-Quievreux 2002, Wang and Guo 2004) and gastropods (Thiriot-Quievreux 2003, Odierna et al. 2006 a, b). Conversely, comparable data on NOR loci and heterochromatin distribution and composition in chitons are only available for *Nuttallochiton mirandus* (Odierna et al. 2008). Two chitons species display quite different patterns of those chromatinic markers. In fact, in *Lepidochitona caprearum* NORs are on the pericentromeric regions of a single pair and in at least three pairs in *Nuttallochiton mirandus*, karyological characters considered, respectively, a primitive and derivate in several taxa, including mollusks (Thiriot-Quievreux 2002, 2003, Wang and Guo 2004, Odierna et al. 2006a, b). Heterochromatin in *Lepidochitona caprearum* is very scarce and with a uniform constitution with the exclusion of that associated with the NOR, which is CMA_3_ positive, then GC rich, as usually observed in several taxa, including mollusks (Odierna et al. 2006a, b, Petraccioli et al. 2010). In contrast heterochromatin in *Nuttallochiton mirandus* is abundant and has a compound composition with clusters AT and GC rich (Odierna et al. 2008). Further studies on localization of NORs and/or heterochromatin composition and distribution in other chitons could provide useful taxonomic and systematic information on this class of mollusks.
